# Effect of Polyvinyl Alcohol on the Rheological Properties of Cement Mortar

**DOI:** 10.3390/molecules25030754

**Published:** 2020-02-10

**Authors:** Fang Liu, Baomin Wang, Yunqing Xing, Kunkun Zhang, Wei Jiang

**Affiliations:** 1 Shaanxi Key Laboratory of Safety and Durability of Concrete Structures, Xijing University, Xi’an 710123, China; liufang_winter@163.com (F.L.); 13892267707@163.com (K.Z.); a18090806090@163.com (W.J.); 2 School of Civil Engineering, Dalian University of Technology, Dalian 116023, China; 18742047452@163.com

**Keywords:** cement mortar, chemical structure, polyvinyl alcohol, rheological properties

## Abstract

Polyvinyl alcohol (PVA) is a kind of water-soluble polymer, which has been widely used in different industries due to its excellent mechanical and chemical properties. In this paper, the effects of polyvinyl alcohol with different hydrolysis and polymerization degrees on the rheological properties of cement mortar are studied. The results show that the rheological properties of PVA-modified cement mortar can be described by the modified Bingham model. The yield stress of modified cement mortar is less than that of unmodified mortar when the degree of polymerization and the content of PVA are small. With the increase of polyvinyl alcohol content and polymerization degree, the yield stress and plastic viscosity of modified cement mortar increase sharply, which are larger than those of the unmodified cement mortar. However, the effect of hydrolysis degree of PVA on yield stress and plastic viscosity of modified cement mortar is not obvious.

## 1. Introduction

Polyvinyl alcohol (PVA) is a kind of water-soluble polymer, which is widely used in different industries due to its excellent mechanical and chemical properties. In the building industry, polyvinyl alcohol is widely used as adhesive [1], modifier [2,3], aggregate-surface pretreatment agent [4,5,6], fiber reinforcement [4,7,8,9,10], etc. Polyvinyl alcohol can be added to cement-based materials in a variety of ways, affecting the properties of cement-based materials. Generally, small amounts of PVA (less than 3%) are added directly to cement-based materials in the form of aqueous solution to improve the properties of cement slurry, mortar, and concrete [3,11,12,13]. The addition of polyvinyl alcohol in aqueous solution to cement-based materials has a great influence on the performance of cement-based materials.

Many studies have shown that the addition of a certain amount of polyvinyl alcohol can improve the workability and water retention of cement-based materials in the state of fresh mixing. Kim et al. [14] proposed the prewetting method to prepare polyvinyl alcohol-modified cement-based materials, and found that the porosity of modified cement-based materials prepared by this method could be reduced to 6%. When a small amount of PVA is added (less than 2%), the air void content and apparent fluidity of fresh mortar and concrete increase, and the bleeding reduces. Due to the increase of fluidity, the slump of the modified concrete increases [3]. Allahverdi et al. [15] studied the effects of different water–cement ratios and polymer–cement ratios on the workability of modified cement mortar. It was found that adding a small amount of polyvinyl alcohol can improve the fluidity of cement mortar. However, with the increase of PVA content, the fluidity of modified cement mortar will be adversely affected. Nguyen et al. [16] studied the effects of molecular weight and dosage of PVA on rheological properties of cement-based materials and found the yield stress and plastic viscosity of cement paste increased with the increasing content and molecular weight of PVA. Shear thinning occurs with the increase of shear rate.

The addition of PVA can delay the hydration of cement and the hydrolysis can affect the hydration process [17,18,19]. Nguyen et al. [17] pointed out that the chemical structure of PVA can affect the hydration process of cement by producing acetate. They also concluded that the quantity of acetate determined the delay time. Georgescu et al. [18] pointed out that the addition of PVA caused the delay of the transformation from AFt to AFm, which delayed the hydration process of cement-based materials.

The physical and chemical properties of polyvinyl alcohol mainly depend on its degree of hydrolysis and polymerization [20]. However, there is still a lack of systematic research on the influence of hydrolysis and polymerization degree of polyvinyl alcohol on the rheological properties of cement-based mortar. The main purpose of this research is to study the effect of PVA on the rheological properties of cement mortar. The materials and test methods used are introduced in Section 2. In Section 3, the modified Bingham model is adopted to analyze the rheological properties of PVA-modified cement mortar. The effects of chemical structure of PVA on the yield stress and plastic viscosity of cement mortar are also discussed. Some main conclusions are drawn in Section 4.

## 2. Raw Materials and Test Methods

### 2.1. Raw Materials

The cement used in this study was P•O 42.5R cement, which was produced by Dalian Onoda Cement Co., Ltd. (Dalian, China). The basic chemical composition and physical properties of the cement are shown in Table 1 and Table 2, respectively. The five types of polyvinyl alcohol selected here were 105, 205, 1799, 1788, and 224, all of which were purchased from Shanghai Macklin Biochemical Co., Ltd. (Shanghai, China) The properties of polyvinyl alcohol are mainly determined by its degree of hydrolysis and polymerization, and Table 3 gives the degree of hydrolysis and polymerization for each polyvinyl alcohol. The sand used here was ISO standard sand, produced by Xiamen ISO Co., Ltd. (Xiamen, China). Experimental water was tap water collected from Dalian, China.

### 2.2. Test Methods

In this paper, the water–cement ratio was 0.4, the sand–cement ratio was 1.5, and the PVA contents were 0%, 0.5%, 1.0%, 1.5%, and 2.0%, by cement mass.

In the preparation of PVA aqueous solution, it was fully swollen and dispersed in water at about 20 °C at first, and then heated in a water bath to different temperatures to accelerate its dissolution. During this process, it was stirred slowly and kept warm for a while until a uniform and transparent solution was formed. The heating time and temperature are shown in Table 4.

The preparation of PVA-modified cement mortar follows: firstly, the mixing vessel and stirring blades were wiped with a wet cloth; secondly, some water was mixed with PVA aqueous solution to a uniform state, and then poured into the mixing vessel; thirdly, cement was added and stirred slowly for 2 min, and then sitting for 1 min; at last, the remaining water was mixed and followed by adding sand slowly, stirring at a low speed for 3 min.

**Figure 1:**
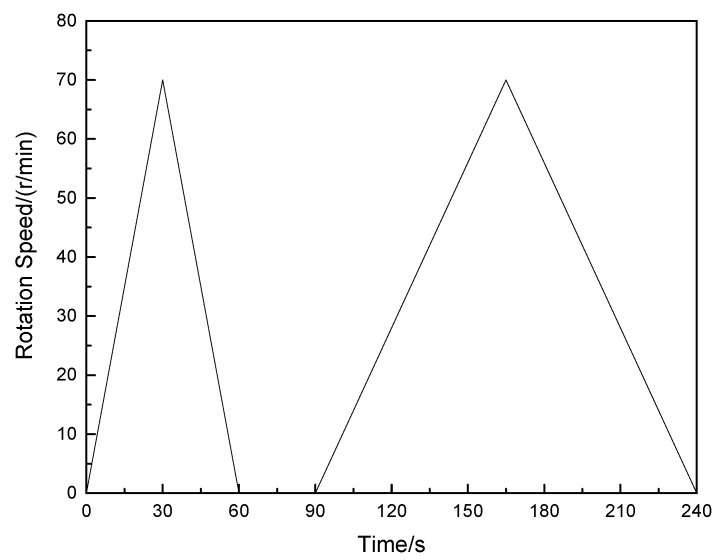
The test procedure.

A rotational rheometer (model RST-SST, manufactured by American Brookfield Company, Shanghai, China) was used to determine the rheological properties of polyvinyl alcohol-modified cement mortar. Figure 1 shows the program for measuring the rheological properties of polyvinyl alcohol-modified cement mortar, which mainly consisted of two stages: preshear and data collection. The purpose of preshearing before data collection was to achieve the same shear state for each group of mortar in the rheological property test.

The test procedure is shown in Figure 1, which included two stages: (a) during preshear, the shear speed increased from 0 to 70 r/min within 30 s, and then decreased to 0 within the next 30 s; (b) in the data collection process, the shear speed increased from 0 to 70 r/min within 75 s, and then decreased to 0 within the next 75 s. During the test, one data point was recorded every second, and a total of 150 data points were obtained, which constituted the up- and down-curves of the shear stress–shear rate.

## 3. Test Results and Discussion

### 3.1. Rheological Model Analysis of PVA-Modified Cement Mortar

**Figure 2:**
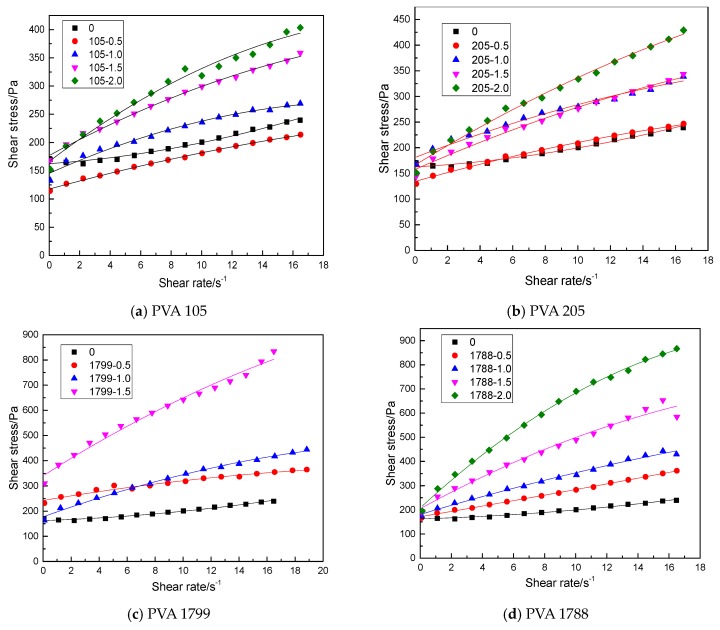

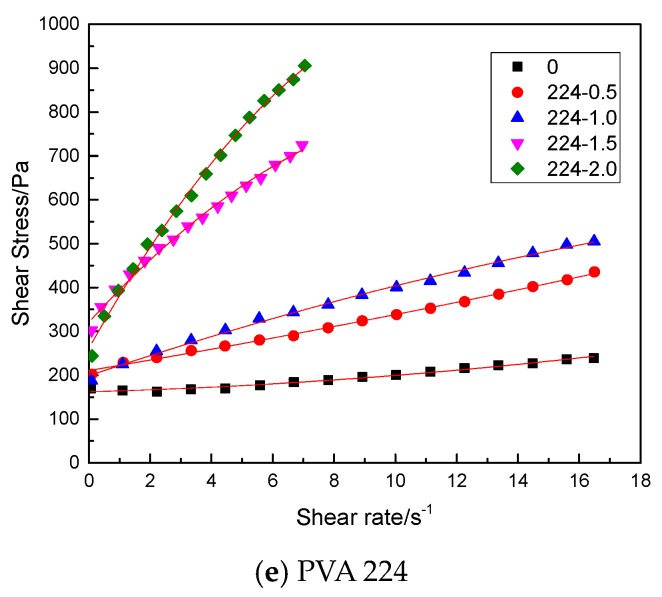
Regression flow curves of different PVA-modified cement mortar: (**a**) PVA 105, (**b**) PVA 205, (**c**) PVA 1799, (**d**) PVA 1788, (**e**) PVA 224.

The rheological data of different PVA-modified cement-based materials were analyzed, as given in Figure 2 and Table 5. In Figure 2, the modified Bingham model was adopted to fit the shear stress–shear rate data of down-curves for PVA-modified cement mortar with different degrees of polymerization and hydrolysis. The number of the fitted data was 16, which were selected equidistant from 75 data points in the down-curve. As can be seen from Figure 2, the modified Bingham model can effectively correlate the shear stress and shear rate data of PVA-modified cement mortar. In addition, it can be found from Figure 2 that rheological models of polyvinyl alcohol-modified mortar with different degrees of hydrolysis and polymerization are basically unchanged, which basically conform to the modified Bingham model [21,22,23]:

(1) $$\tau =\tau_{0}+\mu_{p}\dot{\gamma }+c{\dot{\gamma }}^{2}$$

where τ, $\tau_{0}$, $\mu_{p}$, $\dot{\gamma }$, and *c* are the shear stress, yield stress, plastic viscosity, shear rate, and a regression constant, respectively.

The rheological data of fresh cement mortar with PVA added were fitted with the modified Bingham model, and the regression equation and rheological parameters after fitting are shown in Table 5. As can be seen from Table 5, the fitting coefficient of fresh cement mortar with added PVA is mostly above 0.990, which shows a high correlation and fully indicates that the modified Bingham model has a relatively ideal fitting effect on the mixture. Therefore, the modified Bingham model can be reasonably applied to the study of rheological properties of polyvinyl alcohol-modified cement mortar.

### 3.2. Effect of PVA Chemical Structure on the Yield Stress of Cement Mortar

**Figure 3:**
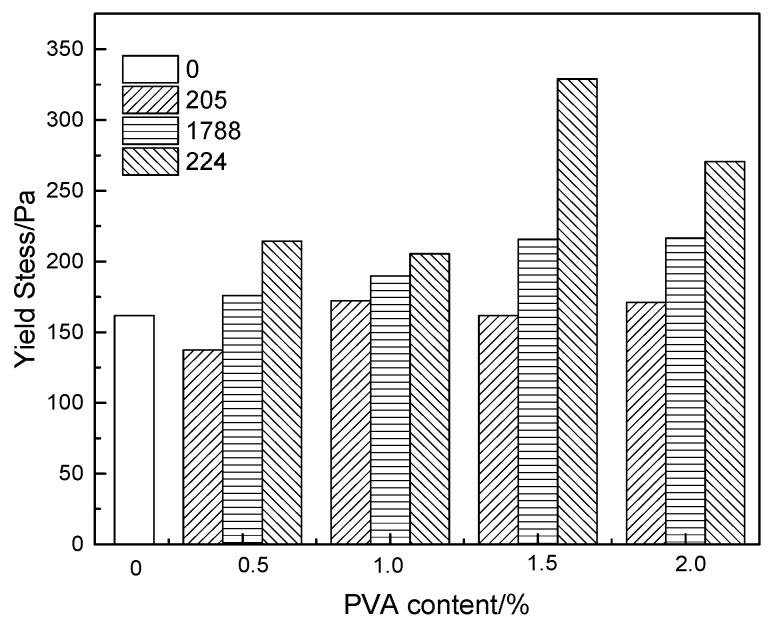
The effect of degree of polymerization and content of PVA on yield stress of cement mortar.

**Figure 4:**
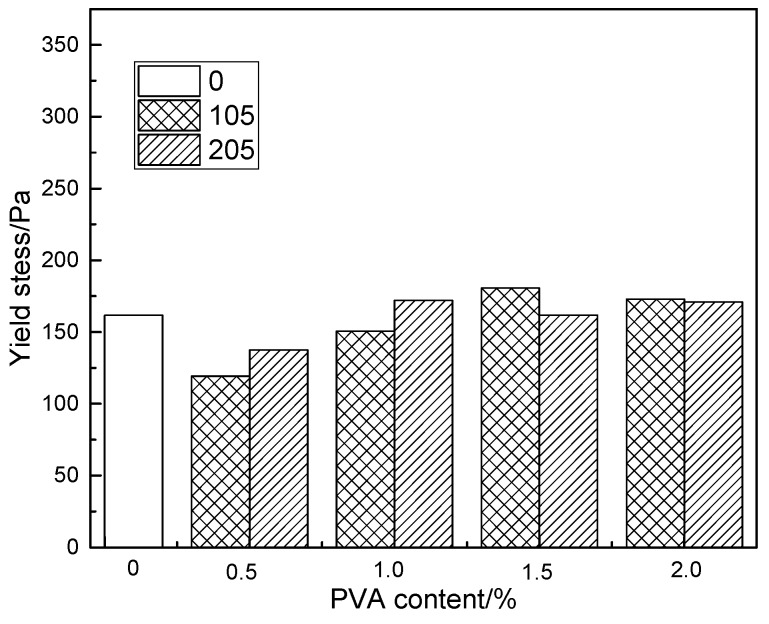
The effect of degree of hydrolysis and content of PVA on yield stress of cement mortar.

Figure 3 and Figure 4, respectively, show the effects of the degree of polymerization, degree of hydrolysis, and content of PVA on the yield stress of fresh modified cement mortar. It can be seen from Figure 3 that with the increase of PVA content, the yield stress of modified cement paste shows a gradually increasing trend. At the same PVA dosage, the yield stress of modified cement paste increases sharply with the increase of polymerization degree. The yield stress of modified cement mortar is less than that of unmodified mortar when the degree of polymerization and the content of PVA are small. However, the yield stress increases obviously with the constant increase of polyvinyl alcohol content and polymerization degree, which is larger than that of the unmodified.

When a small amount of PVA is added, it is adsorbed onto cement and hydrated products. The ball-bearing effect can reduce the friction between cement particles [24,25], resulting in the decrease of the yield stress of modified cement paste. The existence of polyvinyl alcohol can effectively disperse cement-based materials, prevent the generation of flocculation structure, and thus reduce the yield stress of modified cement mortar [24,26]. Due to the surfactant effect of polyvinyl alcohol, some bubbles will be introduced in the stirring process, which to some extent also plays a role in reducing the yield stress [27]. When the dosage of PVA increases continuously, polyvinyl alcohol is not only adsorbed on the surface of cement particles, but also dispersed in the structure of cement mortar relatively independently. The mutual attraction and entanglement of these polyvinyl alcohol molecules increase the friction between particles, thus increasing the yield stress of modified cement mortar [16,28,29]. The higher content of PVA will cause a higher viscosity of PVA aqueous solution, which will increase the viscosity of modified cement paste and further increase its yield stress.

When the content of polyvinyl alcohol is low, polyvinyl alcohol plays a leading role in the dispersion of cement particles, reducing the friction between particles and resulting in the yield stress of modified cement paste being smaller than that of the unmodified. As the PVA content increases, the mutual attraction and entanglement of polyvinyl alcohol molecules among cement particles play a major role in increasing the friction between particles, so that the yield stress of modified paste is greater than that of the reference paste.

The effect of polyvinyl alcohol with different degrees of hydrolysis and dosage on yield stress of fresh modified cement mortar is shown in Figure 4. With the increase of PVA content, the yield stress of modified cement paste increases gradually. A comparison is made between fully hydrolyzed and partially hydrolyzed polyvinyl alcohol-modified cement mortar, from which it can be seen that at the same PVA dosage, the difference between the two yield stresses is not obvious.

### 3.3. Effect of Chemical Structure of PVA on the Plastic Viscosity of Cement Mortar

**Figure 5:**
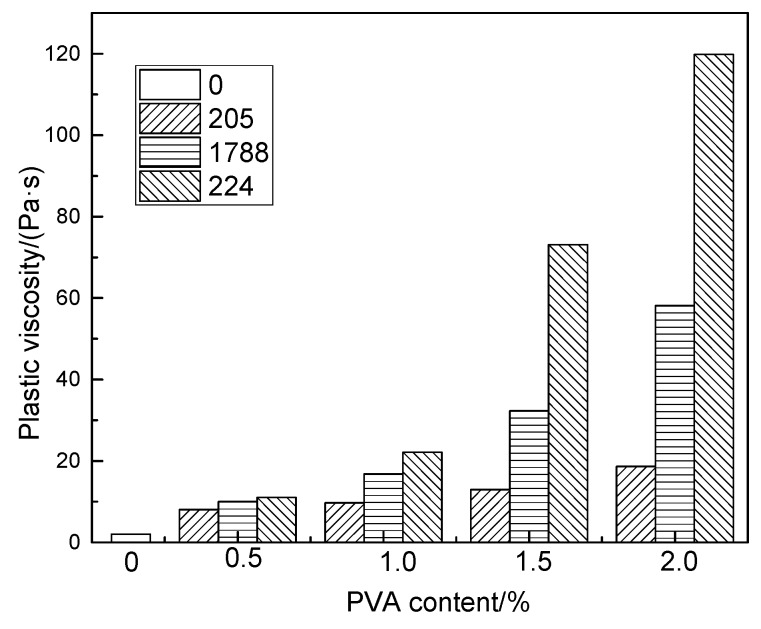
The effect of degree of polymerization and content of PVA on plastic viscosity of cement mortar.

**Figure 6:**
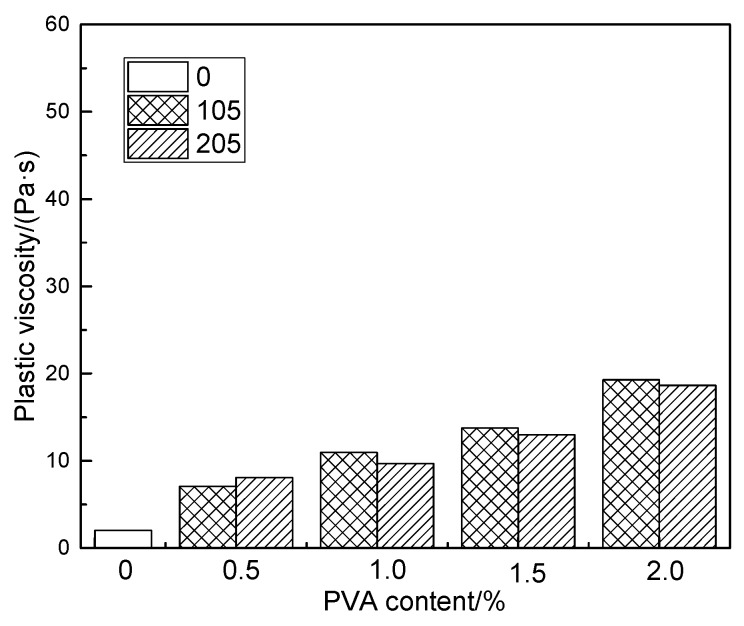
The effect of degree of hydrolysis and content of PVA on plastic viscosity of cement mortar.

Figure 5 and Figure 6, respectively, show the effects of polymerization degree and hydrolysis degree of polyvinyl alcohol on plastic viscosity of fresh modified cement mortar. As can be seen from Figure 5, with the increase of PVA content, the plastic viscosity of modified cement paste increases gradually, which is greater than that of the reference paste. At the same content of PVA, the plastic viscosity increases sharply with the increase of polymerization degree. PVA particles are adsorbed on the surface of cement particles, and intermolecular interactions through hydrogen bonds between hydroxyl groups and cement surfaces form an electrosteric barrier. Under the action of shear force, the electrosteric effect hinders the dissolution of the flocculation structure of cement particles, thereby increasing the plastic viscosity of modified cement mortar [16,24]. On the other hand, the addition of polyvinyl alcohol can increase the viscosity of the aqueous solution inside the cement mortar, thus increasing the plastic viscosity of the modified cement mortar.

With the increase of PVA content, some PVA is adsorbed on the surface of cement mortar particles, while some is dispersed in the matrix pore solution. The interaction between the polyvinyl alcohol molecules distributed in the pore solution further enhances the interaction force between the matrix particles and improves the overall spatial structure of the matrix, thus dramatically increasing the plastic viscosity of the modified cement mortar.

At the same content of PVA, the plastic viscosity of modified cement mortar increases gradually with the increase of the PVA polymerization degree. On the one hand, the viscosity of polyvinyl alcohol solution increases as the degree of polymerization increases, so the plastic viscosity of modified cement mortar increases. On the other hand, due to the increase of PVA polymerization degree, the chain segment of polyvinyl alcohol increases, which further intensifies the interwinding between the chain segments and improves the overall spatial structure of the matrix, thus dramatically increasing the plastic viscosity of modified cement mortar.

Figure 6 shows the effect of hydrolysis degree and content of polyvinyl alcohol on the plastic viscosity of fresh modified cement mortar. It can be found from Figure 6 that with the increase of PVA content, the plastic viscosity of modified cement mortar increases gradually. At the same content of PVA, the plastic viscosity of fully hydrolyzed PVA-modified cement mortar is slightly higher than that of the partially hydrolyzed PVA-modified, in general. Compared with the fully hydrolyzed, in partially hydrolyzed polyvinyl alcohol the intermolecular and intramolecular hydrogen bonds in the chain segment of polyvinyl alcohol are weakened due to the existence of acetoxy, so that partially hydrolyzed polyvinyl alcohol has higher surface activity and lower steric hindrance [30,31]. When adding the same amount of PVA, the interwinding between chain segments of the partially hydrolyzed PVA is less than that of the fully hydrolyzed, and thus the internal flocculation of modified cement mortar is less than that of the fully hydrolyzed, which causes the plastic viscosity of cement mortar modified by partially hydrolyzed PVA to be less than that of the fully hydrolyzed.

Comparing Figure 5 and Figure 6, it can be seen that the change of PVA polymerization degree has a great influence on the plastic viscosity of modified cement mortar. However, the effect of degree of PVA hydrolysis on the plastic viscosity of modified cement mortar is not significant.

## 4. Conclusions

The main purpose of this research was to study the effect of PVA on the rheological properties of cement mortar. Five different types of PVA were used as modifiers and added into cement mortar. The rheological properties of PVA-modified cement mortar were tested. The modified Bingham model was adopted to analyze the rheological properties of PVA-modified cement mortar. The effects of PVA chemical structure on the yield stress and plastic viscosity of cement mortar were also discussed. Based on the experimental results and discussion in this study, the following primary conclusions can be obtained:

The rheological model of polyvinyl alcohol-modified cement mortar conforms relatively well to the modified Bingham model and has a high correlation.

With the increase of PVA content and polymerization degree, the yield stress of modified cement mortar increases sharply. The yield stress of modified cement mortar is less than that of unmodified mortar when the degree of polymerization and the content of PVA are small. The yield stress increases obviously with the constant increase of PVA content and polymerization degree, which is larger than that of the unmodified. However, the effect of hydrolysis degree of PVA on yield stress of modified cement mortar is not obvious.

The plastic viscosity of polyvinyl alcohol-modified cement mortar is sensitive to the changes of PVA polymerization degree and content. With the increase of PVA content, the plastic viscosity of modified cement mortar shows a gradually increasing trend, which is larger than that of the unmodified. With the increase of the degree of PVA polymerization, the plastic viscosity of modified cement mortar increases sharply. Similar to yield stress, the effect of hydrolysis degree of PVA on plastic viscosity of the modified cement mortar is not significant.

## Figures and Tables

**Table 1:** Chemical composition of cement (%).

CaO	SiO_2_	Al_2_O_3_	Fe_2_O_3_	SO_3_	MgO	Na_2_O
61.30	21.43	5.30	2.91	2.51	2.18	0.72

**Table 2:** Physical and mechanical properties of P•O 42.5R cement.

Loss on Ignition/%	Initial Setting Time/min	Final Setting Time/min	Specific Surface Area/m^2^⋅kg^−1^	Flexural Strength/MPa		Compressive Strength/MPa	
3.65	178	240	350	3 d	28 d	3 d	28 d
				6.3	8.2	32.5	54.3

**Table 3:** Degrees of hydrolysis (DH) and degrees of polymerization (DP) of polyvinyl alcohol (PVA) used.

PVA	DP	Molecular Weight	DH
105	500	22,000	99%
205	500	22,000	88%
1788	1700	74,800	88%
1799	1700	74,800	99%
224	2400	105,600	88%

**Table 4:** The heating temperature and time of PVA used.

PVA	Heating Temperature/°C	Time/h
105	70	1.5
205	70	1.5
1788	90	2.5
1799	95	3.0
224	95	3.0

**Table 5:** Rheological parameters of different PVA-modified cement mortar.

Sample	Yield Stress/Pa	Plastic Viscosity/(Pa·s)	Regression Equation	R^2^
0	161.64	2.02	$\tau =161.64+2.02\dot{\gamma }+0.181{\dot{\gamma }}^{2}$	0.984
105-0.5	119.35	7.06	$\tau =119.35+7.06\dot{\gamma }-0.084{\dot{\gamma }}^{2}$	0.997
105-1.0	150.57	10.97	$\tau =150.57+10.97\dot{\gamma }-0.235{\dot{\gamma }}^{2}$	0.991
105-1.5	180.62	13.76	$\tau =180.62+13.76\dot{\gamma }-0.200{\dot{\gamma }}^{2}$	0.997
105-2.0	172.99	19.30	$\tau =172.99+19.30\dot{\gamma }-0.361{\dot{\gamma }}^{2}$	0.986
205-0.5	137.52	8.06	$\tau =137.52+8.06\dot{\gamma }-0.88{\dot{\gamma }}^{2}$	0.997
205-1.0	172.13	9.70	$\tau =172.13+9.70\dot{\gamma }+0.054{\dot{\gamma }}^{2}$	0.998
205-1.5	161.88	12.99	$\tau =161.88+12.99\dot{\gamma }-0.141{\dot{\gamma }}^{2}$	0.994
205-2.0	170.93	18.65	$\tau =170.93+18.65\dot{\gamma }-0.212{\dot{\gamma }}^{2}$	0.995
1799-0.5	249.30	8.03	$\tau =249.30+8.03\dot{\gamma }-0.104{\dot{\gamma }}^{2}$	0.981
1799-1.0	184.10	18.91	$\tau =184.10+18.91\dot{\gamma }-0.287{\dot{\gamma }}^{2}$	0.997
1799-1.5	349.32	34.17	$\tau =349.32+34.17\dot{\gamma }-0.427{\dot{\gamma }}^{2}$	0.988
1788-0.5	175.89	10.01	$\tau =175.89+10.01\dot{\gamma }+0.075{\dot{\gamma }}^{2}$	0.999
1788-1.0	189.71	16.79	$\tau =189.71+16.79\dot{\gamma }-0.052{\dot{\gamma }}^{2}$	0.995
1788-1.5	215.47	32.33	$\tau =215.47+32.33\dot{\gamma }-0.411{\dot{\gamma }}^{2}$	0.983
1788-2.0	216.36	58.13	$\tau =216.36+58.13\dot{\gamma }-1.156{\dot{\gamma }}^{2}$	0.998
224-0.5	214.29	11.02	$\tau =214.29+11.02\dot{\gamma }+0.135{\dot{\gamma }}^{2}$	0.999
224-1.0	205.41	22.16	$\tau =205.41+22.16\dot{\gamma }-0.247{\dot{\gamma }}^{2}$	0.996
224-1.5	328.93	73.14	$\tau =328.93+73.14\dot{\gamma }-0.260{\dot{\gamma }}^{2}$	0.995
224-2.0	270.52	119.85	$\tau =270.52+119.85\dot{\gamma }-4.212{\dot{\gamma }}^{2}$	0.997

Note: No data were obtained when the PVA 1799 dosage was 2.0%, due to the yield stress of fresh modified mortar exceeding the measuring range of the instrument.

## References

[1] Demerlis C.C., Schoneker D.R. (2003). Review of the oral toxicity of polyvinyl alcohol (PVA). Food Chem. Toxicol..

[2] Kim J.H., Robertson R.E. (1998). Effects of polyvinyl alcohol on aggregate-paste bond strength and the interfacial transition zone. Adv. Cem. Based Mater..

[3] Kim J.H., Robertson R.E., Naaman A.E. (1999). Structure and properties of poly(vinyl alcohol)- modified mortar and concrete. Cem. Concr. Res..

[4] Kou S.C., Poon C.S. (2010). Properties of concrete prepared with PVA-impregnated recycled concrete aggregates. Cem. Concr. Compos..

[5] Chai W.W.S., Teo D.C.L., Ng C.K. (2014). Improving the properties of oil palm shell (OPS) concrete using polyvinyl alcohol (PVA) coated aggregates. Adv. Mater. Res..

[6] Mannan M.A., Alexander J., Ganapathy C., Teo D.C.L. (2006). Quality improvement of oil palm shell (OPS) as coarse aggregate in lightweight concrete. Build. Environ..

[7] Ahmed S.F.U., Mihashi H. (2011). Strain hardening behavior of lightweight hybrid polyvinyl alcohol (PVA) fiber reinforced cement composites. Mater. Struct..

[8] Zhang Y., Zhang Z., Liu Z. (2018). Graphite coated PVA fibers as the reinforcement for cementitious composites. Mater. Res. Express..

[9] Hu W., Yang X., Zhou J., Xing H. (2013). Experimental research on the mechanical properties of PVA fiber reinforced concrete. Res. J. Appl. Sci. Eng. Technol..

[10] Zanotti C., Borges P.H.R., Bhutta A., Banthia N. (2017). Bond strength between concrete substrate and metakaolin geopolymer repair mortar: Effect of curing regime and PVA fiber reinforcement. Cem. Concr. Compos..

[11] Mansur A.A.P., Santos D.B., Mansur H.S. (2007). A microstructural approach to adherence mechanism of poly(vinyl alcohol) modified cement systems to ceramic tiles. Cem. Concr. Res..

[12] Singh N.B., Rai S. (2001). Effect of polyvinyl alcohol on the hydration of cement with rice husk ash. Cem. Concr. Res..

[13] Mansur A.A.P., Mansur H.S. (2011). Surface interactions of chemically active ceramic tiles with polymer-modified mortars. Cem. Concr. Compos..

[14] Kim J.H., Robertson R.E. (1997). Prevention of air void formation in polymer-modified cement mortar by pre-wetting. Cem. Concr. Res..

[15] Allahverdi A., Kianpur K., Moghbeli M.R. (2010). Effect of polyvinyl alcohol on flexural strength and some important physical properties of Portland cement paste. Iran. J. Mater. Sci. Eng..

[16] Nguyen D.D., Devlin L.P., Koshy P., Sorrell C.C. (2015). Effect of polyvinyl alcohol on rheology of portland cement pastes. J. Aust. Ceram. Soc..

[17] Nguyen D.D., Devlin L.P., Koshy P., Sorrell C.C. (2016). Effects of chemical nature of polyvinyl alcohol on early hydration of Portland cement. J. Therm. Anal. Calorim..

[18] Georgescu M., Puri A., Coarna M., Viocu G., Voinitchi D. (2002). Thermoanalytical and infrared spectroscopy investigations of some mineral pastes containing organic polymers. Cem. Concr. Res..

[19] Pique T.M., Vazquez A. (2013). Control of hydration rate of polymer modified cements by the addition of organically modified montmorillonites. Cem. Concr. Res..

[20] Thong C.C., Teo D.C.L., Ng C.K. (2016). Application of polyvinyl alcohol (PVA) in cement-based composite materials: A review of its engineering properties and microstructure behavior. Constr. Build. Mater..

[21] Wallevik O.H., Feys D., Wallevik J.E., Khayat K.H. (2015). Avoiding inaccurate interpretations of rheological measurements for cement-based materials. Cem. Concr. Res..

[22] Nehdi M., Rahman M.A. (2004). Estimating rheological properties of cement pastes using various rheological models for different test geometry, gap and surface friction. Cem. Concr. Res..

[23] Feys D., Wallevik J.E., Yahia A., Khayat K.H., Wallevik O.H. (2013). Extension of the Reiner-Riwlin equation to determine modified Bingham parameters measured in coaxial cylinders rheometers. Mater. Struct..

[24] Betioli A.M., Gleize P.J.P., John V.M., Pileggi R.G. (2012). Effect of EVA on the fresh properties of cement paste. Cem. Concr. Compos..

[25] Чepкинcкий, Ю·C (1984). Polymer Cement Concrete.

[26] Moukwa M., Youn D., Hassanali M. (1993). Effects of degree of polymerization of water soluble polymers on concrete properties. Cem. Concr. Res..

[27] Zhang C., Yuan X., Zhou S. (2004). Effect of mineral admixtures and superplasticizer on rheological performance of cement based materials. Concrete.

[28] Liang N. (1995). Mechanism analysis of polymer modified cement and cement concrete. J. Xi’an Hwy. Univ..

[29] Zheng S., Niu K., Tian B., Chen L., Cheng Z., Xu Y. (2017). Effect of Polymer Latex on Rheological Properties of Cement Mortar. J. Build. Mater..

[30] Marten F.L., Kroschwitz J.I., Howe-Grant M. (2002). Vinyl alcohol polymers. Kirk-Othmer Encyclopedia of Chemical Technology.

[31] Yu Z. (1982). Study on foaming mechanism of partially alcoholized polyvinyl alcohol in solution. Cotton Text. Technol..

